# Genome-Based Analysis of Genetic Diversity, Antimicrobial Susceptibility, and Virulence Gene Distribution in *Salmonella* Pullorum Isolates from Poultry in China

**DOI:** 10.3390/ani14182675

**Published:** 2024-09-14

**Authors:** Yiluo Cheng, Jigao Zhang, Qi Huang, Qingping Luo, Tengfei Zhang, Rui Zhou

**Affiliations:** 1 State Key Laboratory of Agricultural Microbiology, College of Veterinary Medicine, Huazhong Agricultural University, Wuhan 430070, China; chengyiluo@webmail.hzau.edu.cn (Y.C.); qhuang@mail.hzau.edu.cn (Q.H.); 2 Key Laboratory of Prevention and Control Agents for Animal Bacteriosis (Ministry of Agriculture and Rural Affairs), Hubei Provincial Key Laboratory of Animal Pathogenic Microbiology, Institute of Animal Husbandry and Veterinary, Hubei Academy of Agricultural Sciences, Wuhan 430064, China; 17533321144@163.com (J.Z.); qingping0523@163.com (Q.L.)

**Keywords:** *Salmonella enterica* serovar Pullorum, antibiotic resistance, antibiotic-resistance gene, virulence gene, antigenic type

## Abstract

**Simple Summary:**

*Salmonella enterica* serovar Pullorum (*S*. Pullorum) is a formidable pathogen within the poultry industry and constitutes a substantial threat to global poultry production. Pullorum disease eradication and antibiotic treatment are two main strategies for controlling this disease. Therefore, understanding the prevalence and resistance characteristics of *S*. Pullorum is important for its control. The objective of this study was to determine the antigenic types and genotype characteristics of *S*. Pullorum isolates from China, and to clarify the relationship between antibiotic resistance and resistance genes. In this study, all the isolates tested were of standard antigenic types, with ST92 the predominant genotype, and 92.5% of the isolates displayed multidrug resistance. Our analysis of virulence genes indicated that the isolates expressed numerous factors associated with secretion systems. These data provide valuable information for the prevention and control of *S*. Pullorum infections in the poultry industry.

**Abstract:**

Pullorum disease, caused by *Salmonella enterica* serovar Pullorum (*S*. Pullorum) infection, is a major pathogenic threat to the poultry industry. In this study, 40 *S*. Pullorum isolates from seven provinces of China were comprehensively analyzed in terms of antigenic type and antimicrobial susceptibility, and their drug-resistance genes and virulence genes were identified with whole-genome sequencing (WGS). We show that all these isolates were standard antigenic types, with ST92 the predominant genotype (92.5%). Disk diffusion assays revealed high resistance rates to streptomycin (92.5%), ciprofloxacin (82.5%), and ampicillin (80%), and the resistance rates to streptomycin, gentamicin, ampicillin, and cefotaxime were higher in isolates from sick chickens than in those from healthy chickens. In addition, *gyrA* mutations and eight acquired resistance genes were identified, with *aac(6′)-Iaa* the most prevalent, followed by *blaTEM1β*, *sul2*, and the GyrA S83F mutation. The resistance phenotypes to streptomycin, ampicillin, and ciprofloxacin correlated strongly with the presence of the *aac(6′)-Iaa* resistance gene, *blaTEM1β* resistance gene, and *gyrA* mutations, respectively. Analysis of the virulence genes showed that the isolates expressed numerous factors associated with secretion systems, including SPI-1 and SPI-2. Overall, this study extends our understanding of the epidemiology and antibiotic resistance of *S*. Pullorum in China.

## 1. Introduction

*Salmonella enterica* serovar Pullorum (*S*. Pullorum) is an important pathogen of poultry and poses a serious threat to the poultry industry [1]. Chickens less than 3 weeks old are the most common group infected by *S.* Pullorum [2]. Infection is characterized by white, sticky diarrhea and acute sepsis, and the morbidity and mortality rates are very high, as the mortality rate for young chickens can reach 100%. Infected adult chickens may be asymptomatic, but infection reduces production efficiency and the bacteria can be vertically transmitted through contaminated eggs [3]. Therefore, the eradication of Pullorum disease from breeding hens is the main strategy for controlling this disease [4]. Antibiotics use is also an important treatment method for infected chickens. However, Pullorum disease remains a serious problem worldwide, especially in some developing countries, severely hampering the poultry industry and causing substantial economic losses [5,6].

*Salmonella* Pullorum belongs to *Salmonella* serogroup D1 (O:9). It is characterized by the antigenic profile O:1,9,12 and further subdivided into standard and variant antigenic types based on differences in the O12 antigenic factor [7,8]. Knowing the antigenic type is important for its serological diagnosis. In the United States, the prevalence of variant isolates of *S*. Pullorum has been as high as one-third in the early 19th century [9]; however, another study analyzed 150 strains isolated between 1990 and 1991 and only four variant strains were found [10]. As far as we know, the molecular mechanisms regulating the differentiation of standard and variant strains remain unclear. The prevalence of *S*. Pullorum is markedly low in some developed countries, which is attributed to Pullorum disease eradication strategies and robust vaccination programs [1]. However, the positive rate of Pullorum disease is still high in China. When Song et al. investigated *Salmonella* across 17 poultry-breeding farms located in various provinces of China, they identified a positive infection rate of 3.59% [11].

As well as eradication and biosecurity measures, the use of antibiotics is crucial for the treatment of salmonellosis in chickens. The long-term use of antibiotics in poultry farming offers some benefits, including reducing bacterial infections and stimulating the growth of chickens [12]. However, it has also led to the development of bacterial resistance. Alarmingly, Li et al. (2022) reported that 91.43% of *Salmonella* strains were resistant to ciprofloxacin, and that resistance to ampicillin was similarly high, at 71.43% [13]. In Bangladesh, 95% of isolates of *Salmonella enterica* serovars Typhi and Paratyphi showed resistance to azithromycin [14]. Infections caused by antibiotic-resistant bacteria can result in treatment failure and present a significant challenge to public health.

Whole-genome sequencing (WGS), which emerged with the development of molecular biological techniques, is a cost-effective and efficient approach that significantly extends our understanding of the genetic determinants underlying antibiotic resistance and virulence in microbial strains. The data obtained from such analyses are also a crucial resource for global research institutions, supporting further investigations and providing substantial support for the prevention and control of *Salmonella* outbreaks [15,16]. In this study, we investigated the antigenic types, genetic diversity, antibiotic resistance, and the distributions of virulence and resistance genes of *S.* Pullorum isolated from chickens in China. Our goal was to extend our understanding of the local prevalence of *S.* Pullorum in China.

## 2. Materials and Methods

### 2.1. Isolation and Antigenic Type Analysis of S. Pullorum Isolates

The strains were collected from sick or healthy-looking chickens in Anhui, Guangxi, Henan, Hubei, Jiangsu, Shandong, and Zhejiang Provinces of China. To isolate the strains, the samples collected were pre-enriched in 10 mL of buffered peptone water (HOPEBIO, Qingdao, China). After incubation for 12 h at 37 °C, 0.2 mL of each culture was transferred into 10 mL of Rappaport–Vassiliadis medium (HOPEBIO). The selective enrichment cultures were then transferred to a Salmonella Shigella Agar medium (HOPEBIO) and incubated at 37 °C for 18 h. Suspected isolates were then confirmed with PCR amplification of the *glgc* gene using the specified primers: SGP-F, 5′-cggtgtactgcccgctat-3′, SGP-R, and 5′-ctgggcattgacgcaaa-3′ [17]. The antigenic types were identified with the single-factor serum agglutination method, with the standard and the variant sera (China Institute of Veterinary Drug Control, Beijing, China). Strains CVCC 519 and CVCC 530 were used as controls. The former is a standard antigenic type of strain, and the latter is a variant antigenic type of strain.

### 2.2. Antimicrobial Susceptibility Test

The Mueller–Hinton agar (HOPEBIO) disk diffusion method was used, according to the Clinical and Laboratory Standards Institute (CLSI) method, to evaluate the antimicrobial susceptibility of the 40 *Salmonella* strains. Nine antibiotics from six categories were used in this experiment: macrolides (erythromycin, ERY), aminoglycosides (streptomycin and gentamicin), β-lactams (ampicillin and cefotaxime), sulfonamides (sulfamethoxazole and trimethoprim–sulfamethoxazole), tetracyclines (tetracycline), and quinolones (ciprofloxacin). Isolates producing a bacteriostatic ring within the intermediate range of an antimicrobial agent were classified as resistant for analytical purposes, and multidrug resistance (MDR) was defined as resistance to at least three different antimicrobial classes. *Escherichia coli* ATCC 25922 was used as the quality control strain.

### 2.3. DNA Extraction and WGS

The genomic DNA from all 40 *Salmonella* test specimens was extracted from cultures incubated overnight in Luria–Bertani (LB) broth with the TaKaRa MiniBEST Bacterial Genomic DNA Extraction Kit Ver. 3.0 (Takara Bio Inc., Dalian, China), according to the instructions provided. The DNA was then quantified with the NanoDrop™ One spectrophotometer (Thermo Fisher Scientific, Wilmington, DE, USA). Libraries were constructed from the extracted genomic DNA and sequenced with the Illumina NovaSeq 6000 platform (MajorBio Co., Shanghai, China), which generated reads of 2 × 150 bp in length. After sequencing, the raw reads were filtered with the fastp software (version 0.19.6) to obtain clean reads by eliminating adapter sequences and low-quality reads (Q < 20). The clean reads were assembled with SOAPdenovo version 2.04. A phylogenetic tree was constructed based on core single-nucleotide polymorphisms (SNPs), and MINTyper 1., accessed on 1 January 2024) was used to analyze all the *Salmonella* isolates tested for SNP sequences. The MEGA 6.06 program was used to construct the phylogenetic tree.

### 2.4. Bioinformatic Analysis

Virulence genes were predicted based on the Virulence Factor Database (VFDB) (http://www.mgc.ac.cn/VFs/main.htm, accessed on 13 April 2024), which contained four major bacterial VF categories (i.e., adhesion and invasion, secretion system, toxin, and iron acquisition) [18]. The tool ResFinder v.4.1 (http://genepi.food.dtu.dk/resfinder, accessed on 13 April 2024) was used to detect acquired antimicrobial resistance (AMR) genes and point mutations in specific genes conferring AMR, with 90% minimum percentage identity and 60% minimum length coverage used as the selection criteria. The assembled contigs were submitted to the MLST 2.0 website to determine their sequence types (https://cge.food.dtu.dk/services/MLST/, accessed on 13 April 2024).

### 2.5. Correlation Analysis of Susceptibility Phenotypes and Genotypes

We analyzed the genome sequencing results to statistically examine the known resistance genes and specific point mutations present in the *S.* Pullorum isolates. These data were then combined with the antibiotic sensitivity test results for each strain to investigate the potential relationship between the resistance phenotype of *Salmonella* in chickens and the genotype predicted with WGS. The probability of a resistance gene or point mutation was determined by dividing the number of occurrences of a particular gene or point mutation by the total number of isolated strains. The presence of one or more resistance genes or point mutations indicated potential resistance to a specific class of antibiotics. Consistency between the phenotype and genotype was seen in strains that were phenotypically and genotypically positive, or phenotypically and genotypically negative. A lack of consistency was observed in strains that were phenotypically positive but genotypically negative, or phenotypically negative but genotypically positive.

### 2.6. Identification of Gtr Operons within S. Pullorum Genomic Sequences

Two methods were used to detect the presence of the *gtr* gene cluster in the complete genomic sequences of the *S.* Pullorum isolates. The first method involved uploading the FASTA file containing the genomic sequencing data of the *S.* Pullorum isolates to the National Center for Biotechnology Information (NCBI). Comparison of the data with the published *Salmonella gtr* gene cluster allowed the identification of homologous sequences [19]. Alternatively, the *gtrC* gene from bacteriophage P22 was used to identify *Salmonella* genomic sequences with BLASTn. Sequences were classified as *gtr* if their *gtrC* homologue had adjacent *gtrB-* and/or *gtrA*-like sequences.

## 3. Results

### 3.1. Genetic Diversity and Antigenic Type Analysis

A total of 40 *S*. Pullorum strains (15 strains isolated from healthy chickens and 25 strains isolated from sick chickens) were analyzed and sequenced (BioProject accession number: PRJNA1138287). These *S.* Pullorum isolates were differentiated into two sequence types (STs) based on multilocus sequence typing (MLST). ST92 was the dominant genotype (92.5%, 37/40), whereas the other three isolates belonged to ST2151 (Table 1).

**Figure 1:**
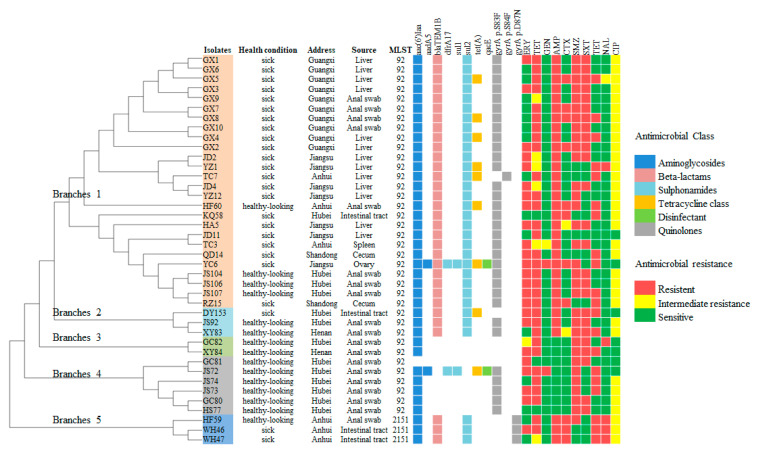
Genetic relationships, antimicrobial-resistance phenotypes, and the distribution of resistance-related genes determined in this study. The phylogenetic tree was constructed based on genomic SNPs to clarify the evolutionary relationships between strains. Genetic determinants of antibiotic resistance were systematically categorized according to their corresponding antibiotic classes and visually differentiated with color coding. Isolates clustered on five distinct branches of the phylogenetic tree, each marked by a unique color to facilitate differentiation: red (branch 1), light blue (branch 2), green (branch 3), gray (branch 4), dark blue (branch 5). STR, streptomycin; GEN, gentamicin; AMP, ampicillin; CTX, cefotaxime; SMZ, sulfamethoxazole; SXT, trimethoprim–sulfamethoxazole; TET, tetracycline; NAL, nalidixic acid; CIP, ciprofloxacin; ERY, erythromycin.

On a phylogenetic tree constructed based on SNPs, the isolates were grouped into five main branches with the three ST2151 strains clustered on one separated branch (Figure 1). The serum agglutination tests showed that the CVCC 519 agglutinated with standard serum, and the CVCC 530 agglutinated with variant serum. All of the isolates in this study agglutinated with standard serum but not variant serum, identifying them as standard antigenic type strains.

### 3.2. Antimicrobial Susceptibility Analysis

The disk diffusion method was used to test the susceptibility of these isolates to nine antibiotics. As shown in Table 2, high resistance rates were observed to streptomycin (92.5%), ciprofloxacin (82.5%), and ampicillin (80%), with lower resistance rates to erythromycin (62.5%), trimethoprim–sulfamethoxazole (62.5%), and tetracycline (50%). In contrast, resistance to gentamicin was seen in only 10% of strains.

Further analysis revealed that the resistance rates to streptomycin, gentamicin, ampicillin, and cefotaxime were higher in isolates from sick chickens than in those from healthy chickens. Notably, the resistance rate to ampicillin in sick chickens was 100%. Surprisingly, isolates from healthy chickens showed higher resistance rates to sulfamethoxazole, tetracycline, and erythromycin.

In total, 27 different AMR patterns were identified (Table 3). Of the 40 isolates, 92.5% (37/40) showed resistance to three or more antimicrobial categories, indicating multidrug resistance (MDR). The commonest MDR patterns observed were streptomycin-ampicillin-ciprofloxacin in 70% (28/40) of strains, followed by streptomycin-sulfamethoxazole-ciprofloxacin in 62.5% (25/40) of strains and streptomycin-ampicillin-sulfamethoxazole in 60% (24/40) of strains.

### 3.3. Antibiotic-Resistance Genes and Resistance Mutations

The WGS analysis identified eight acquired resistance genes and three *gyrA* mutations as responsible for resistance to six classes of antibiotics or disinfectants (Figure 1). The concordance between phenotypic and genotypic antimicrobial resistance is shown in Table 4.

The isolates contained two aminoglycoside antibiotic-resistance genes: *aac(6′)-Iaa* and *aadA5*. The gene *aac(6′)-Iaa* was detected in 39 isolates (97.5%), whereas *aadA5* was only found in two isolates. Combinations of resistance genes were strongly associated with streptomycin resistance (92.5%, 37/40) but not significantly associated with gentamicin resistance (10%, 4/40).

One β-lactam resistance gene, *blaTEM1β*, was detected in 32 isolates (80%), and each of these 32 isolates was resistant to ampicillin, whereas the other eight *blaTEM1β*-negative isolates were ampicillin susceptible. Therefore, ampicillin resistance seemed to be 100% associated with the presence of *blaTEM1β*. In contrast, 32.5% (13/40) of the isolates carrying *blaTEM1β* showed resistance to cefotaxime.

The isolates we examined contained three sulfonamide-resistance genes, *dfrA17*, *sul1*, and *sul2*. Gene *sul2* was detected in 32 isolates (80%), whereas *sul1* and *dfrA17* were only detected in two isolates. The correlation between sulfonamide resistance and the resistance genes in the isolated strains was slightly more consistent, with a correlation of 62.5% (25/40) for sulfamethoxazole and 50% (20/40) for trimethoprim–sulfamethoxazole.

Among the 40 isolates tested, nine (22.5%) contained the *tet(A)* gene, all of which were resistant to tetracycline. Moreover, 11 strains without the *tet(A)* resistance genes were also resistant to tetracycline.

Of the total strains, 90% (36/40) carried a *gyrA* point mutation. Specifically, 32 of the strains had the GyrA S83F mutation, one strain had the GyrA S84F mutation, and three strains had the GyrA D87N mutation. Of these isolates, 82.5% (33/40) of those with a *gyrA* mutation showed intermediate-level resistance to ciprofloxacin, whereas the isolates with none of these three mutations were sensitive to ciprofloxacin. Therefore, the correlation between the resistance phenotype and the *gyrA* mutations was strong.

Neither 23S point mutations nor genes conferring macrolide resistance were detected in our isolates. However, the erythromycin-resistance rate was high, at 62.5%. Furthermore, two strains carried the *qacE* gene for disinfectant resistance.

### 3.4. Detection of Virulence Genes

**Figure 2:**
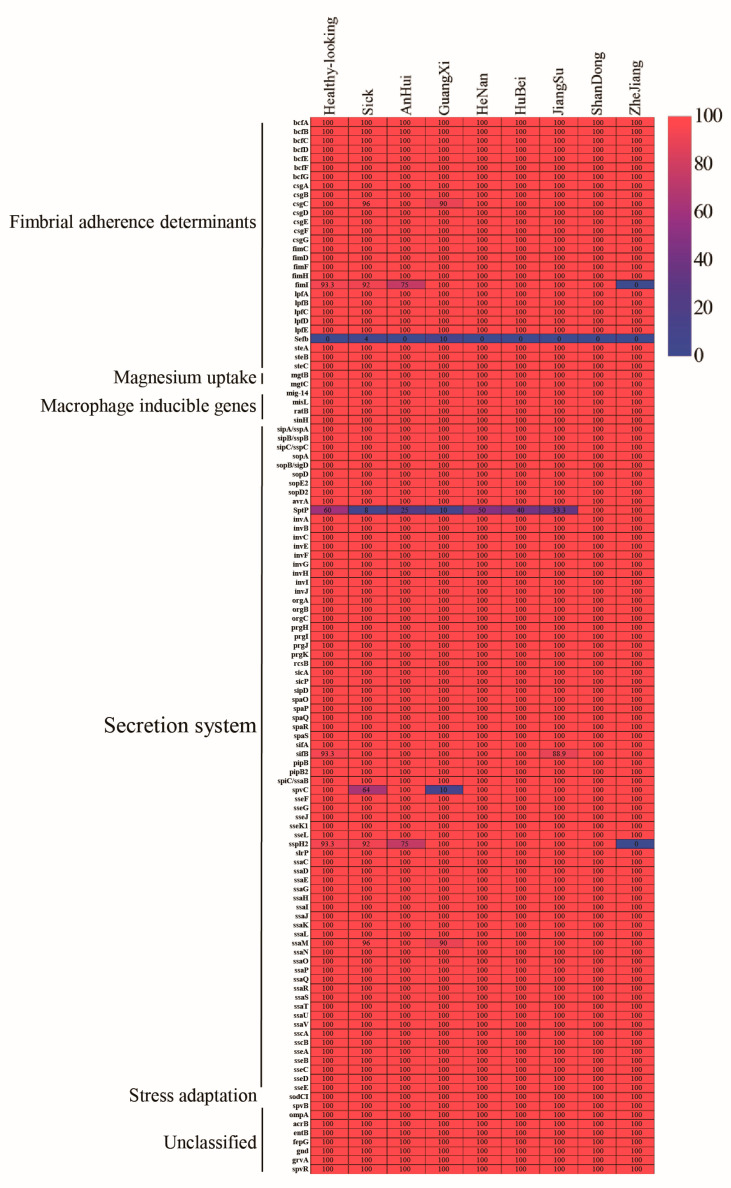
Distribution of virulence genes among the isolates studied. Colors of the individual cells vary with the percentage prevalence of each virulence gene.

Based on the VFDB, a total of 117 virulence-related genes, involved in fimbrial adherence, secretion systems, stress adaptation, toxins, and so on, were identified (Figure 2). Among these, 109 genes were consistently present across all isolates from both healthy and sick chicken flocks: 69 genes associated with secretion systems (most of which were located in *Salmonella* virulence island 1 [SPI-1] and SPI-2); 25 fimbrial adherence genes; two magnesium uptake genes; one macrophage-inducible gene; one stress adaptation gene; one toxin gene; and 10 unclassified genes. Genes belonging to the Spv family enhance the ability of *Salmonella* strains to grow inside the endothelial cells of the reticulate system. The *spvR* and *spvB* genes were present in all the strains, whereas the *spvC* gene was found in 77.5% of strains. The gene encoding the type III secretion system effector protein SptP, which is crucial for the infection process of *Salmonella*, was identified in only 27.5% of the strains. Interestingly, *sptP* and *spvC* were more prevalent in *S.* Pullorum strains from healthy chicken flocks than in those from unhealthy flocks; only the *sifB* gene exhibited a higher carrier rate in the sick chicken flocks. The fimbriae-synthesis-related gene *sefB* was only present in one sick chicken. The macrophage-inducing gene *mig-14* was detected in all strains.

### 3.5. Gtr Operon Analysis

The operon *gtrABC* is reported to encode the glycosyltransferase for O-antigen glucosylation in *S.* Typhimurium [20]. We examined whether the presence of *gtrABC* is associated with the antigenic-type development of *S.* Pullorum. A BLAST analysis revealed that the isolates contained *gtr* operons of varying lengths, designated *gtrABC*^1^ (2552 bp), *gtrABC*^2^ (2739 bp), and *gtrABC*^3^ (2919 bp). Specifically, all isolates belonging to ST2151, one isolate belonging to ST92, and the CVCC 530 strain (variant antigenic type) contained *gtrABC*^1^ and *gtrABC*^3^, whereas all the other isolates (ST92) and the CVCC 519 strain (standard antigenic type) contained all three *gtrABC* operons (Table 5). 

## 4. Discussion

The impact of Pullorum disease, caused by *S*. Pullorum infection, is particularly significant in the poultry industry. Therefore, to comprehensively understand the antibiotic resistance and virulence of *Salmonella* strains in poultry, we used WGS together with meticulous phenotypic analyses of 40 *S.* Pullorum isolates sourced from poultry in China.

In this study, MLST was used to investigate the genetic diversity of *S*. Pullorum, which was compared across different regions [21]. Previous research has demonstrated a strong correlation between sequence type and serovar [22]. The analysis of MLST patterns based on WGS revealed that the majority of *S*. Pullorum isolates (37/40) belonged to ST92, consistent with previous research [5,11], indicating that ST92 is the most prevalent genotype. Three strains were identified as ST2151, with only minor mutations in the *hemD* gene relative to ST92, implying that ST2151 probably developed from ST92 through small-scale evolutionary changes. Song et al. identified one strain of *S*. Pullorum ST470 in 126 *Salmonella* isolates [11], but ST470 was not detected in the present study. *Salmonella* infections in poultry have a long history, and ST92 may have evolved to readily colonize the chicken body and may have coevolved with poultry. Wilson et al. used a DNA fingerprinting analysis to analyze the antigenic types of 150 strains of *S*. Pullorum and identified 50 intermediate strains and four variant strains [10]. However, after single-factor serum detection, the strains isolated in the present study were all standard antigenic types, with no intermediate or variant types, indicating that standard *S*. Pullorum strains are the dominant antigenic type in China. It is unclear whether there is a relationship between different STs and the *S*. Pullorum antigenic types. The glycosyltransferase enzyme Gtr is reported to modify the O antigen of *Salmonella*, assisting the bacterium to evade the host’s immune system and so enhancing its virulence and infectivity [23]. All our isolates contained 2–3 *gtrABC* operons, but the roles of *gtrABC* in both O antigen modification and virulence are unclear.

Animals are considered a significant source of drug-resistant pathogens, so antibiotic resistance is a serious issue for the poultry industry and a threat to public health [24]. In this study, we evaluated the susceptibility of *S.* Pullorum isolates to nine antibiotics in six categories. High resistance rates to streptomycin (92.5%), ciprofloxacin (82.5%), and ampicillin (80%) were observed. Quinolone resistance is a common problem in China and other countries [25,26], and it is noteworthy that sick chickens had a higher resistance rate to quinolones and β-lactams than did healthy chickens. In the past, quinolones were frequently provided as a feed supplement [27], and ampicillin is widely used in poultry production [5], which may exert selective pressure on bacterial strains, with the ultimate emergence of MDR *Salmonella* strains. Of the isolates tested, 92.5% were resistant to at least three different types of antibiotics (and were therefore considered MDR). This percentage is higher than that reported by Song et al. in isolates from nine provinces of China [11], in diseased birds from Northern China [28], and in dead-in-shell chicken embryos from Shandong Province, China [29]. β-Lactam and quinolone antibiotics are the first-line options for the clinical treatment of *Salmonella* infections in humans. Therefore, it is crucial to develop strict policies to regulate the use in poultry farming of antibiotics that are potentially used by humans. It is noteworthy that two *S.* Pullorum strains from healthy chickens from different sources displayed resistance to each of the six types of antibiotics. This could potentially compromise antibiotic treatments for infected chickens. Our results have significant implications for the public health system.

To investigate the mechanisms underlying the phenotypic resistance of our isolates, WGS was used to detect the presence of various antibiotic-resistance genes. The analysis revealed the presence of 11 different genes encoding resistance to six antimicrobial classes and one disinfectant. The *aac(6′)-Iaa* gene, which is responsible for aminoglycoside resistance, was found in 97.5% of the isolates examined. This proportion is similar to that reported in previous research conducted in China and South Korea [30,31]. Previous studies have demonstrated that plasmid-mediated quinolone resistance and amino acid mutations in the quinolone-resistance-determining region (QRDR) of the DNA gyrase are commonly present in quinolone-resistant *Salmonella* strains, contributing to varying levels of fluoroquinolone resistance [25]. None of the *S*. Pullorum isolates in our study carried the *qnrA*, *qnrB*, *qnrC*, *qnrD*, *qnrS*, or *aac-Ib* gene. Therefore, the observed quinolone resistance in this study may be attributable to point mutations in the QRDR. Our analysis showed that 90% of the strains displayed S83F, S84F, or D87N substitutions in the GyrA enzyme, reducing their susceptibility (intermediate) to quinolones, consistent with previous research [13]. The consistency observed between phenotypic and genotypic resistance was relatively strong, particularly for ampicillin and ciprofloxacin (Table 4). In contrast, the consistency between phenotypic and genotypic resistance to erythromycin or tetracycline was only moderate. Our results are consistent with those for *Campylobacter* strains isolated in central China and reported by Xiao et al. [32]. Phenotypic resistance to an antibiotic can sometimes be due to mechanisms such as coresistance or cross-resistance, even when no specific genetic element is detected. Conversely, not all identified genes necessarily confer phenotypic resistance [16,33,34].

The pathogenicity of *S*. Pullorum is considered to be multifactorial, involving various genes, such as SPI-1 and SPI-2, that act as virulence factors, eliciting a host immune response and significantly contributing to the virulence of the bacterium. Our isolates contained a substantial number of genes associated with SPI-1 and SPI-2. These genes encode two distinct type III secretion systems (T3SS), which are critical for the pathogenicity of *Salmonella* [35]. SPI-1 primarily facilitates the initial invasion of the intestinal epithelial cells by the bacterium and is most active during the early phase of infection [36]. Conversely, SPI-2 is pivotal for the survival and replication of *Salmonella* within host macrophages. This system becomes activated once the bacterium has infiltrated the host cells, assisting in its evasion of the host’s defenses and in establishing an optimal environment for replication [37]. Previous studies have shown that the SEF14 fimbriae are essential for the full virulence of *S.* Enteritidis in vivo [38]. The *sef* operon is located on a small pathogenicity island and contains four structural genes (*sefABCD*) required for the translocation and biogenesis of the SEF14 fimbriae. The *sefACD* genes were not detected in any isolate in this study, and the *sefB* gene was detected in only one strain. Zhou et al. reported that fimbrial degradation improves a strain’s adaptability to macrophages [39]. As a result, it is hypothesized that *S*. Pullorum strains are gradually losing *sefABCD* and other fimbrial genes during their evolution, leading to a reduced inflammatory response and enhanced survival of the bacterium in the host. The *sifB* gene could play a significant role in infection within intestinal epithelia or Peyer’s patches [40,41]. However, considering *sifB* may function analogously to *sifA* or *sseJ*, and given that both aforementioned genes exhibit a uniform carrier rate of 100% in both healthy and sick chickens, the variance in solitary *sifB* gene carriage might not be sufficient to influence the strain’s pathogenicity. It is noteworthy that the prevalence of the *sptP* gene was higher in healthy chickens than in diseased chickens. Lin et al. demonstrated that SptP plays a vital role in suppressing the activation of the mitogen-activated protein kinase pathway and reducing the secretion of tumor necrosis factor alpha from infected J774A.1 macrophages [42]. The importance of SptP to virulence in vivo was also demonstrated by the reduced intestinal colonization of chicks, pigs, and cattle when *sptP* was disrupted in S. Typhimurium ST4/74 [43]. We hypothesize that SptP facilitates the latent infection of strains, allowing them to more effectively colonize tissues such as the intestine and to evade clearance by macrophages.

In this study, all isolates examined were the standard antigenic type, and variant antigenic type strains were not identified, likely attributable to the limited sample size and insufficient coverage. Moreover, our efforts to discern differences in gtrABC modification between standard and variant antigenic types were impeded by the absence of variant antigenic isolates.

## 5. Conclusions

In this study, ST92 was the dominant genotype in our *S*. Pullorum isolates, and all the isolates were standard antigenic type strains. Our results also showed that 92.5% of isolates were multidrug resistant, and the *blaTEM1β*, *aac(6’)-Iaa* genes and gyrA mutations were closely associated with ampicillin, streptomycin, and ciprofloxacin resistance, respectively. An analysis of virulence genes revealed that the isolates expressed numerous factors associated with secretion systems. Notably, the prevalence of the *sifB* gene was marginally higher in isolates from sick chickens compared to those from healthy ones, whereas *sptP* and *spvC* were more prevalent in isolates from healthy chickens. These results provide important epidemiological and genomic information for the control of *S*. Pullorum in poultry. 

## Figures and Tables

**Table 1:** Antigenic types and MLST types of *S*. Pullorum isolates in this study.

Antigenic Type	MLST Pattern (No. of Isolates)	arcO	dnaN	hemD	hisD	purE	sucA	thrA
Standard	ST92 (37)	5	2	3	7	31	41	11
Standard	ST2151 (3)	5	2	361	7	31	41	11

**Table 2:** Resistance rates of the studied *S*. Pullorum isolates to nine antibiotics.

Antibiotic Category	Antimicrobial Agents	Healthy-Looking (*n* = 15)		Sick (*n* = 25)		Total (*n* = 40)	
		No. of Resistant Isolates ^a^	Resistance Rates (%)	No. of Resistant Isolates	Resistance Rates (%)	No. of Resistant Isolates	Resistance Rates (%)
Aminoglycoside	STR	13	86.7%	24	96.0%	37	92.5%
	GEN	1	6.7%	3	12.0%	4	10.0%
β-lactams	AMP	7	46.7%	25	100.0%	32	80.0%
	CTX	3	20.0%	10	40.0%	13	32.5%
Sulphonamide	SMZ	15	100.0%	17	68.0%	32	80.0%
	SXT	10	66.7%	15	60.0%	25	62.5%
Tetracyclines	TET	8	53.3%	12	48.0%	20	50.0%
Quinolones	CIP	11	73.3%	22	88.0%	33	82.5%
Macrolides	ERY	11	73.3%	14	56.0%	25	62.5%

^a^ esistant isolates’ includes resistant and intermediate isolates.

**Table 3:** Resistance patterns of the 40 *S.* Pullorum isolates.

Resistance Patterns	Number
STR-AMP	1
ERY-SXT-SMZ	1
SXT-SMZ-CIP	1
AMP-CTX-TET-CIP	1
ERY-STR-SXT-SMZ	1
STR-AMP-TET-CIP	1
STR-SMZ-TET-CIP	1
ERY-STR-AMP-SMZ-CIP	1
ERY-STR-AMP-TET-CIP	2
ERY-STR-SXT-SMZ-CIP	1
ERY-STR-SXT-SMZ-TET	1
ERY-STR-SMZ-TET-CIP	1
STR-AMP-CTX-TET-CIP	1
STR-AMP-SXT-SMZ-CIP	3
ERY-STR-GEN-SMZ-TET	1
ERY-STR-AMP-CTX-TET-CIP	2
ERY-STR-AMP-SXT-SMZ-CIP	6
ERY-STR-AMP-SXT-SMZ-TET	1
STR-AMP-CTX-SXT-SMZ-CIP	1
STR-AMP-CTX-SMZ-TET-CIP	1
STR-AMP-SXT-SMZ-TET-CIP	1
ERY-STR-AMP-CTX-SXT-SMZ-CIP	2
ERY-STR-AMP-CTX-SMZ-TET-CIP	1
ERY-STR-AMP-SXT-SMZ-TET-CIP	1
ERY-STR-GEN-AMP-SXT-SMZ-CIP	2
STR-AMP-CTX-SXT-SMZ-TET-CIP	3
ERY-STR-GEN-AMP-CTX-SMZ-TET	1

**Table 4:** Concordance (percentages) between phenotypic and genotypic antimicrobial resistance.

Antimicrobial Agent	Genotype(+) Phenotype(−)	Genotype(−) Phenotype(+)	Genotype(−) Phenotype(−)	Genotype(+) Phenotype(+)
STR	5% (2)	0% (0)	2.5% (1)	92.5% (37)
GEN	87.5% (35)	0% (0)	2.5% (1)	10% (4)
AMP	0% (0)	0% (0)	20% (8)	80% (32)
CTX	47.5% (19)	0% (0)	20% (8)	32.5% (13)
SMZ	20% (8)	17.5% (7)	0% (0)	62.5% (25)
SXT	32.5% (13)	12.5% (5)	5% (2)	50% (20)
TET	0% (0)	27.5% (11)	50% (20)	22.5% (9)
CIP	7.5% (3)	0% (0)	10% (4)	82.5% (33)
ERY	0% (0)	62.5% (25)	37.5% (15)	0% (0)

**Table 5:** *gtr* operons detected in *S*. Pullorum isolates.

Sources of Strains	Antigenic Types	No.	Sequence Types	*gtrABC*^1^ (2552 bp)	*gtrABC*^2^ (2739 bp)	*gtrABC*^3^ (2919 bp)
Isolates	Standard	36	ST 92	√	√	√
	Standard	1	ST 92	√	×	√
	Standard	3	ST 2151	√	×	√
CVCC 519	Standard	1	ST 92	√	√	√
CVCC 530	Variant	1	ST 92	√	×	√

## Data Availability

The datasets presented in this study can be found in online repositories (BioProject accession number: PRJNA1138287).
